# Impact of European Bison Grazing (*Bison bonasus* (L.)) on Species and Functional Traits of Carabid Beetle Assemblages in Selected Habitats in Poland

**DOI:** 10.3390/biology10020123

**Published:** 2021-02-05

**Authors:** Axel Schwerk, Daniel Klich, Elżbieta Wójtowicz, Wanda Olech

**Affiliations:** 1 Institute of Environmental Engineering, Department of Landscape Art, Warsaw University of Life Sciences–SGGW, 02-787 Warsaw, Poland; 2 Institute of Animal Sciences, Department of Animal Genetics and Conservation, Warsaw University of Life Sciences–SGGW, 02-787 Warsaw, Poland; daniel_klich@sggw.edu.pl (D.K.); wanda_olech@sggw.edu.pl (W.O.); 3 Kobiór Forest District, State Forests, 43-211 Piasek, Poland; elzbieta.wojtowicz@katowice.lasy.gov.pl

**Keywords:** Carabidae, European bison, functional traits, grazing, biological diversity, habitat management

## Abstract

**Simple Summary:**

Currently, we are observing a drastic decline in insect biodiversity. The aim of this study was to determine whether grazing by European bison (*Bison bonasus* (L.)) has potential for the implementation of conservation measures. Therefore, a study on a free-ranging European bison population and captive herds in enclosures was carried out in order to determine whether this animal’s grazing activity impacts species composition and the ecological characteristics of carabid beetle assemblages. No notable influence on the numbers of individuals of carabid beetles could be detected, but there was an indication that high-intensity grazing may cause an increase in the number of species. Increased grazing activity had a stronger influence on the ecological characteristics of the species assemblages than on the species composition. This result indicates that using European bison grazing as a method for nature conservation may have more potential in regulating properties and functions of the ecosystem than in the conservation of specific species or species assemblages of carabid beetles.

**Abstract:**

Currently we are observing a drastic decline in insect fauna on a large scale. Grazing is regularly used as an ecological method of protecting or restoring special biotopes that are important for species conservation. The European bison (*Bison bonasus* (L.)) is the largest living wild terrestrial animal in Europe; therefore, a large impact on flora and fauna as a result of its grazing activity can be assumed. There might be potential for implementing conservation measures that employ active grazing. Therefore, a study on a free-ranging European bison population and captive herds in enclosures was carried out in order to determine whether European bison grazing has an impact on carabid beetle assemblages and whether the degree of this impact (if any) depends on the intensity of grazing. No notable influence on numbers of individuals of carabid beetles could be detected, but there was an indication that high-intensity grazing may cause an increase in the number of species. Increased intensity of grazing seems to have only a weak impact on the species assemblage structure, but it has a stronger impact on the composition of functional traits in the assemblage, as demonstrated in particular by the significant impact of captive herds. The stronger relation between grazing intensity and the functional traits of the carabid beetle assemblages than between grazing intensity and assemblage structure indicates that using European bison grazing as a method of ecological engineering in the context of nature conservation may have more potential in regulating properties and functions of the ecosystem than in the conservation of specific species or species assemblages of carabid beetles.

## 1. Introduction

Currently, more than 1 million insect species have been described [1]. They play an important role in ecosystems; for example, as elements of food webs. However, currently, we are observing a drastic decline in insect fauna on a large scale [2,3,4,5,6]. There is a need to develop strategies and guidelines to counteract this process, but in order to do so we need to understand the reasons for the observed species decline and the response of insect populations to ecological species-protection measures.

Grazing is regularly used as an ecological method in order to protect or restore special biotope types that are important for species conservation [7]; for example, protecting sedges and mosses in the Biebrzanski National Park in Poland using cattle and horse grazing [8] or the recovery of *Calluna* heather in The Netherlands [9]. Carabid beetles are suitable for investigations on insect population trends because they are a species-rich group of insects that are ubiquitous in the majority of terrestrial ecosystems [3]. They efficiently reflect environmental variation and bear indicator potential at various spatial scales [10]. Carabid beetles react quickly to management practices in grasslands and forests, including grazing [11]. Hence, carabid beetles can be useful indicators for the assessment of grazing as a species-conservation measure. Previous studies have shown that the degree of grazing intensity can variously impact insect populations [12,13,14]. Heavy grazing by cattle and horses in Welsh peatland resulted in a strong influence on carabid assemblages whereas light sheep grazing had little impact [12]. Researchers [14] who investigated the response of carabid beetles to grazing in Cretan shrublands described them as good indicators of grazing pressure at assemblage level, rather than having direct impacts on specific species. Overgrazing resulted in lower species richness, and species richness and diversity were maximal under moderate to relatively high levels of grazing.

The European bison (*Bison bonasus* (L.), also called wisent) is the largest living wild terrestrial animal in Europe. It is still an endangered species that requires comprehensive conservation efforts [15]. For this reason, the research conducted so far has focused mainly on its biology and threats to species conservation and population development [16,17,18,19]. In turn, little research has been done that can explain the effect of the European bison on ecosystems [20,21]. To our knowledge, no research has been done on the effects of this species on carabid beetles. Because of European bison’s grazing activity, a strong impact on flora and fauna can be assumed. This is important because the population is still growing and new areas are being populated as a result of conservation activities [22,23]. Hence, due to increasing grazing pressure, an increasing population also impacts the landscape and populations of individual species, including various insect species. This increasing activity might pose some threats, but it also offers potential for implementing conservation measures through the use of active grazing.

The aim of the study was to assess the impact of European bison grazing on carabid fauna in forest and meadow ecosystems. This was carried out in two variants: (a) based on a free-ranging population (Augustowska Forest) and (b) based on a captive population (enclosures in the Kobiór Forest District and Niepołomice Forest District). Based on these two experiments, we wanted to test the hypotheses that (1) European bison grazing has an impact on the carabid beetle assemblages, and (2) the degree of this impact depends on the intensity of grazing by European bison.

## 2. Materials and Methods

### 2.1. Study Sites and Field Methods

#### 2.1.1. Study Sites–Free-Ranging Population

**Figure 1:**
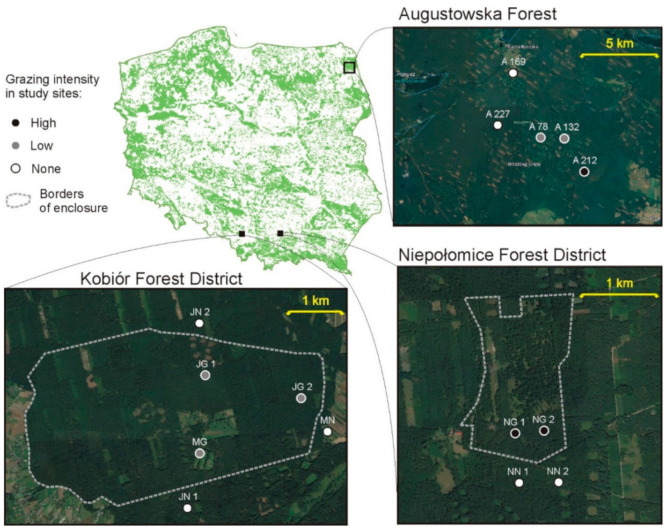
Location of the study sites (circles) in Augustowska Forest (A—study sites), the Kobiór Forest district (JN—grazed forest sites, JN—non-grazed forest sites; MG—grazed meadow; MN—non-grazed meadow) and the Niepołomice Forest District (NG—grazed sites; NN—non-grazed sites).

Augustowska Forest is located in the northeastern part of Poland (Figure 1). It is one of the largest forest complexes in Poland, covering an area of approximately 1140 km^2^. The main habitats are coniferous and mixed coniferous forests with strong dominance of Scots pine. For the purposes of the study, five mid-forest meadows at a distance of 0 to 8 km from the European bison acclimatization enclosure were selected. The first assessment of the carabid beetle assemblages was conducted in 2017, before the animals were released into the wild. The second assessment was performed in 2019, after the animals were released.

In March 2018, as part of a European bison conservation project [24], a herd of 8 European bison was released into the wild, after which their presence in the area was monitored using a GPS collar. The collar allowed the position of the herd to be determined every 4 h during the day. The presence of European bison in individual meadows was assessed on the basis of the location points transmitted by the collar. Kernel density estimation was performed in QGIS software (version 3.4.5, Madeira). We performed Kernel 99% for the home range assessment of the herd, thanks to which we delimited the occupied area. The core area, i.e., the area of intensive use, was determined on the basis of Kernel 50%. In this way, individual meadows were categorized according to the intensity of their use by European bison. Meadows located in Kernel 50% were marked as high grazing intensity; meadows in Kernel 99% were marked as low grazing intensity; the meadows out of the home range area (Kernel 99%) were marked as non-grazed.

#### 2.1.2. Study Sites–Captive Herds

The study sites in the two enclosures were located in southern Poland (Figure 1). Mixed coniferous forests with high dominance of Scots pine is the main forest habitat in both enclosures. Two meadow sites (one grazed, one non-grazed) and four forest sites (two grazed, two non-grazed) were located in the “Jankowice” enclosure (about 740 ha with over 40 individuals), which is situated in the Kobiór Forest District (south of Katowice). The non-grazed forest habitats were similar in habitat, stand species composition and fertility. Grazing intensity was about 0.06 individuals per ha. The “Niepołomice” enclosure (about 70 ha with over 20 individuals), which is situated in the Niepołomice Forest District (east of Kraków), had four forest sites (two grazed, two non-grazed). Grazing intensity was about 0.4 individuals per ha. We used the term “grazing intensity”, but we realize that the influence of bison is more comprehensive: apart from eating plants, they also fertilize and trample the ground [25]. The non-grazed forest habitats were similar in habitat, stand species composition and fertility. The habitats varied in terms of the age of the main stand, and a corresponding non-grazed habitat of the same age was designated for each grazed habitat (85, 110 or 120 years). All analyzed habitats defined as “non-grazed” were located outside but near the enclosure area (up to 50 m); habitats defined as “grazed” were located inside the enclosure area and the European bison had unlimited access to them. Individual habitats were assigned according to their intensity of use by European bison, i.e., forests inside the “Niepołomice” enclosure were marked as high grazing intensity; forests inside the “Jankowice” enclosure were marked as low grazing intensity; meadow and forest outside enclosures were marked as non-grazed.

#### 2.1.3. Carabid Beetle Trapping

Carabids were collected using pitfall traps [26]. A total of five traps were set about 5 m apart at each site. The traps were glass jars topped with a funnel (upper diameter of about 10 cm, height about 6.5 cm) set flush with the soil surface. A roof was suspended a few centimeters above the funnel and min. 96% ethylene glycol was used as a killing agent and preservative. Carabids were sampled from mid-May to mid-September. The traps were collected and replaced at intervals of about two weeks (eight trapping intervals).

Determination and nomenclature of the individuals collected was carried out according to literature [27].

### 2.2. Data Arrangement

Except for the rarefaction analyses (see Section 2.3), for each study site the catches of the five traps were pooled. For each species, the total number of individuals per study site and the dominance value (percentage share of the individuals of the respective species on the total number of individuals collected at the study site) were calculated.

We selected the following functional traits of carabids for analyses of composition of functional traits in the assemblages: habitat preference, dispersal power, trophic specialization, and moisture preference. These traits are important for understanding the changes in assemblage characteristics due to grazing. Regarding habitat preference, the collected species were divided into forest species, eurytopic species with a wide range of habitats, and species that are characteristic of open areas. The dispersal power is very important for species in the colonization and recolonization of habitats. We grouped the species according to whether they had high dispersal power (ability to fly) or low dispersal power (inability to fly). The species were divided with respect to their trophic specialization into big zoophages, small zoophages, and hemizoophages (species with mixed or preferably vegetable food). Carabid beetles are known to react to changes in moisture conditions. We divided our species into hygrophilous, mesophilous, and xerophilous species. Classification of the species to the respective groups was based on the literature [27,28,29,30,31,32,33,34,35,36,37].

For each of the studied sites, a grazing level of either none (0: no grazing), low (1: weak grazing activity at Augustowska forest complex, grazed sites in “Jankowice” enclosure) or high (2: intensive grazing activity at Augustowska forest complex, grazed sites in “Niepołomice” enclosure) was defined.

### 2.3. Statistical Analyses

PAST v. 4.03 (Oslo, Norway) was used to carry out Mao’s tau sample rarefaction in order to evaluate sampling efficiency [38]. Each individual trap at every trapping interval was used as a sample. As some traps were damaged, 40 samples were not obtained in all cases. In particular, several of the traps in the meadows in the “Jankowice” enclosure were damaged by wild boar and/or European bison. In the “Niepołomice” enclosure, the traps at study sites 38, 39 and 41 could not be collected during the seventh trapping interval; thus, the last collection covered the seventh and eighth interval.

We applied indirect gradient analyses in order to illustrate the major carabid beetle assemblage structure patterns at the study sites. In order to determine the impact of grazing intensity in relation to the other variables (year of the study, area type, study area), direct gradient analyses were carried out using Monte Carlo permutation tests (unrestricted, 1999 permutations) and automatic forward selection of variables (reduced model) [39].

Detrended Correspondence Analyses (DCA) and Detrended Canonical Correspondence Analyses (DCCA) were first used to select the appropriate statistical model based on the longest gradient [40]. In the case of short gradients (in our study < 2.0) Principal Components Analyses (PCA) and Redundancy Analyses (RDA) were used; in the case of long gradients (in our study > 3.0), Correspondence Analyses (CA) and Canonical Correspondence Analyses (CCA) were selected. PCA and RDA were carried out with scaling focused on inter-sample distances and no post-transformation of species scores and CA and CCA were carried out using inter-sample distance scaling and Hill’s scaling and un-weighted data for each species. Because dominance values (percentage share of the respective number of individuals of a species or a functional trait) were used, the data were not transformed.

The following environmental variables were included in the direct gradient analyses: grazing intensity (“Intensity”) was included in both experimental parts with values 0, 1, 2; year (“2017” or “2019”) was included in the Augustowska forest complex with the year of the study as dummy variables; habitat type (“Forest” or “Meadow”) and area (“Jankowice” or “Niepołomice”) were included in the experiment on captive herds as dummy variables.

All ordination analyses were carried out using Canoco for Windows 4.56 and CanoDraw for Windows 4.14 [39,41].

## 3. Results

### 3.1. Free Ranging-Population

**Figure 2:**
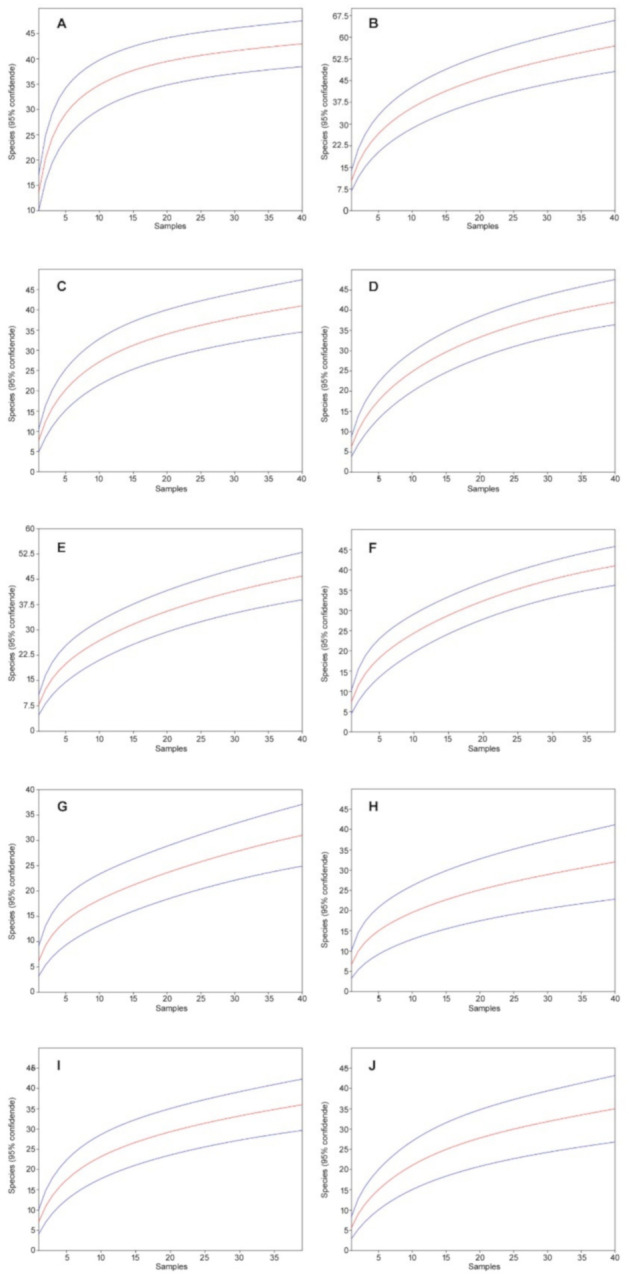
Mao’s tau sample rarefaction curves (red lines) with 95% confidence intervals (blue lines) for the study site in Augustowska forest: (**A**) A212. 2017, (**B**) A212, 2019, (**C**) A78, 2017, (**D**) A78, 2019, (**E**) A132, 2017, (**F**) A132, 2019, (**G**) A169, 2017, (**H**) A169, 2019, (**I**) A227, 2017, (**J**) A227, 2019 (95% confidence intervals are indicated).

In 2017, we collected 5356 individuals from 79 species; in 2019, we collected 6056 individuals from 78 species. Accordingly, in both years combined, we collected 11,412 individuals from 93 species (Table 1). All rarefaction curves, both in 2017 and 2019, are close to flatten out and converge against a number approximate to the number of species detected by us in the individual study sites (Figure 2).

The results did not show a relation between grazing intensity and the number of collected individuals. However, a relation between grazing intensity and the number of collected species is suggested. In 2019 (after the release of European bison), the highest number of species was collected at study site A212 (high grazing intensity); moderate numbers were collected at study sites A78 and A132 (low grazing intensity); the lowest numbers were collected at study sites A169 and A227 (no grazing). In 2017, the data were similar for the study sites without grazing or with low intensity grazing, but they were far lower for the study site with high intensity grazing compared to 2019 (Table 1).

**Figure 3:**
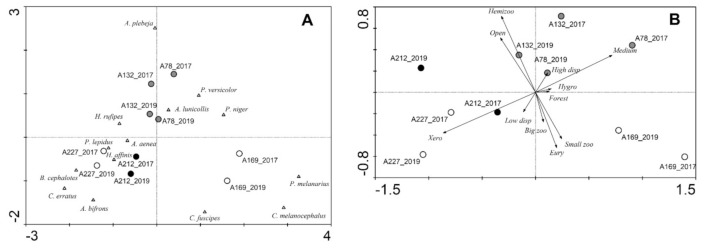
Ordination plots for the study sites (circles; grazing intensity: black—high, grey—low, white—none) and species (triangles) in Augustowska Forest: (**A**) CA based on carabid beetle assemblages, (**B**) PCA based on the functional traits of carabid beetle assemblages.

In the CA based on the species assemblages (Figure 3A) the first and second ordination axes explained 29.7 and 24.1% of the variation in the dataset, respectively. In the diagram, the assemblages from the two study years are located close to one another, but the assemblages of the year 2019 are somewhat shifted to the bottom of the diagram. The assemblages at the grazed sites are located close to the center of the diagram. Grazed sites seemed to be characterized more by species that prefer open areas and dry conditions, such as *Amara aenea* and *Harpalus rufipes*. The first and second ordination axis of the PCA based on functional traits (Figure 3B) explained 64.8 and 18.1% of the variation in the dataset, respectively. The PCA diagram also shows the tendency that both years of the individual study sites are located close to each other. Sites in the year 2019 are shifted to the left side of the diagram. This shift is by trend more strongly pronounced for the grazed sites. Regarding the functional traits, the xerophilous are directed towards the left side of the diagram.

Both grazing intensity and year of study did not show a statistically significant impact on species assemblages or functional traits (Table 2 and Table 3). However, in contrast to the results regarding species assemblages, the impact of grazing intensity on functional traits was stronger than the impact of the year of the study.

### 3.2. Captive Herds

**Figure 4:**
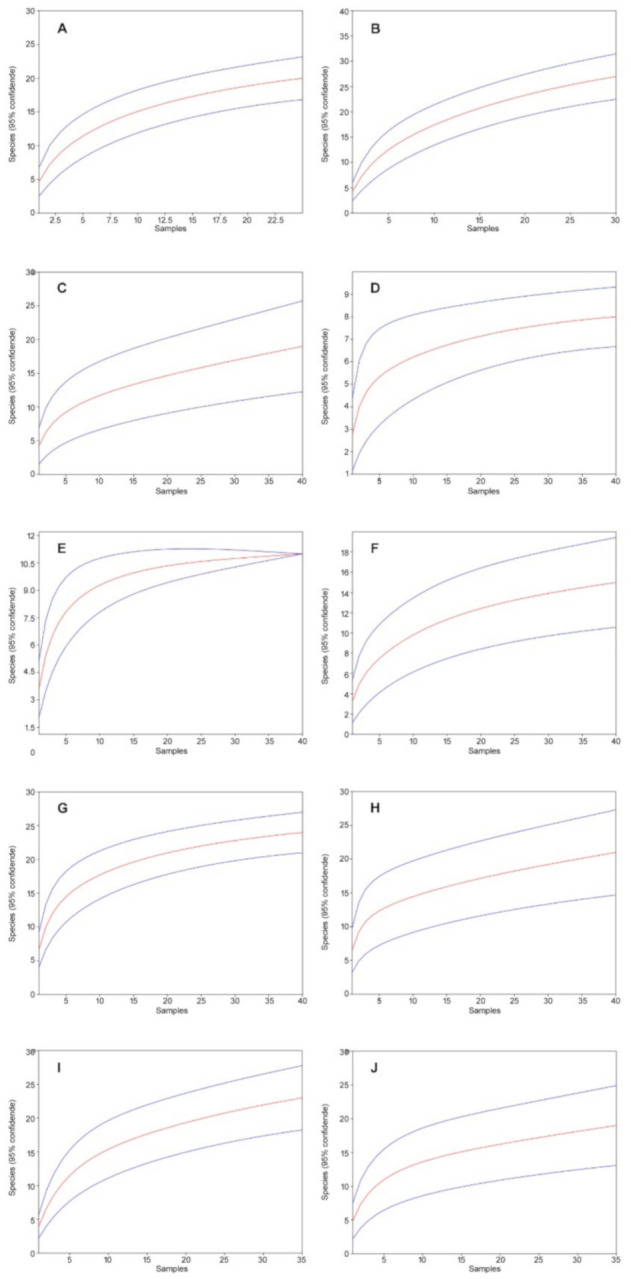
Mao’s tau sample rarefaction curves (red lines) with 95% confidence intervals (blue lines) for the study sites in the Kobiór Forest district and the Niepołomice Forest District: (**A**) MG, (**B**) MN, (**C**) JG1, (**D**) JN1, (**E**) JG2, (**F**) JN2, (**G**) NG1, (**H**) NN1, (**I**) NG2, (**J**) NN2 (95% confidence intervals are indicated).

In 2018, a total of 9615 individuals from 60 species were collected (Table 4). The rarefaction curves are close to flatten out and converge for all study sites against a number approximate to the number of species detected in the study; study site JG2 explicitly flattened out and reached the respective value (Figure 4).

Additionally, due to trap losses, the meadows showed generally low numbers of individuals, but the species number was higher on the non-grazed site MN compared to the site with low-intensity grazing (MG). Regarding the forest sites, no clear trend with respect to the numbers of individuals could be observed. However, the highest species numbers were collected at study sites NG1 and NG2 in the “Niepołomice” enclosure, both of which were under high-intensity grazing. Both sites had higher numbers of species than their corresponding non-grazed sites. In the “Jankowice” enclosure, study site JG1 contained much more species than the corresponding site JN1, but study site JG2 contained less species than the corresponding site JN2 (Table 4).

**Figure 5:**
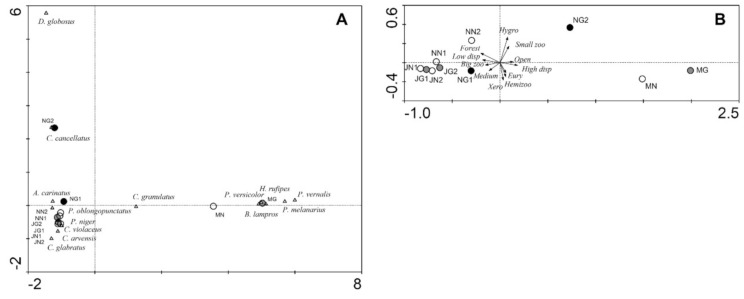
Ordination plots for the study sites (circles; grazing intensity: black—high, grey—low, white—none) and species (triangles) in the Kobiór Forest district and the Niepołomice Forest District: (**A**) CA based on carabid beetle assemblages, (**B**) PCA based on the function altraits of carabid beetle assemblages

In the CA based on the species assemblages (Figure 5A), the first and second ordination axes explained 45.9 and 22.2% of the variation in the dataset, respectively. In the diagram, the meadows are shifted to the right along the first ordination axis far from the forest sites. The heavily grazed sites NG1 and NG2 are shifted somewhat along the second axis to the top of the diagram. Species located close to meadows were characteristic of open areas and are able to fly, such as *Bembidion lampros* and *Harpalus rufipes*, whereas forest species are located on the left side of the diagram. *Carabus cancellatus* and particularly *Dyschirius globosus* are shifted to the upper part of the diagram. In the PCA based on functional traits (Figure 5B), the first and second ordination axes explained 95.2% and 2.6% of the variation in the dataset, respectively. In the ordination diagram, the meadows are also shifted to the right along the first ordination axis far from the forest sites. The heavily grazed sites (particularly NG2) are shifted towards the meadows. Both of the lightly grazed sites (JG1, JG2) are shifted slightly to the right side of the diagram when compared to the respective corresponding site. Functional traits directed towards the right side of the diagram are preference for open areas and high dispersal power.

Habitat type and area had a statistically significant impact on the species assemblages (Table 5). Grazing intensity did not show a significant impact, but there was a trend (*p* = 0.076). However, regarding the functional traits, habitat type and grazing intensity showed a significant impact (Table 6).

## 4. Discussion

The rarefaction analyses suggested that the species numbers detected in the study for the individual study sites were sufficiently reliable. Our results did not show a notable influence on numbers of individuals of carabid beetles. However, there is an indication that high-intensity grazing may cause an increase in species numbers. Increased grazing intensity seems to have only a weak impact on species assemblage structure, but it has a stronger impact on the composition of functional traits in assemblages, as demonstrated in particular by the significant impact in the captive herds.

Our study revealed a more pronounced impact of other factors on the composition of the carabid beetle assemblages, which may mask the impact of grazing. Not surprisingly, a main factor was habitat type, but location also had a strong impact on the species assemblage structure. The year of the study did not have a significant impact. Researchers [42] who have studied the impact of low-intensity cattle grazing detected a higher amount of variance that was explained by habitat and year than by grazing. However, grazing intensity is a notable factor. This result is in accordance with former studies [12]. Researchers [43] showed that on heather moorland in northeast Scotland the carabid distribution was strongly influenced by the organic content of soil and the height of *Calluna*, but heavy grazing levels had a significant impact on the biomass, height and shoot structure of *Calluna* and resulted in a significant change in carabid assemblages. In our study the relative impact of grazing intensity in relation to the other variables also increased with increasing grazing intensity. Mowing affects ecosystems in a similar way and has been proposed as a surrogate for grazing [13]. It has been shown that mowing intensity is related to the degree of changes in carabid beetle assemblages [44].

It is worth noting that the observed impact of grazing intensity in our study more strongly affected the composition of carabid functional traits compared to the species assemblage structure. This is an important detail, because functional traits are important with respect to the contribution of the species to ecosystem properties and services and the resilience of the ecosystem. The vulnerability of the ecosystem properties and benefits may be affected by changes in functional traits of the species [45]. For subalpine grasslands, it was also shown that mammalian grazing not only altered the composition of carabid assemblages, but it also caused functional trait shifts [46]. In differently managed grasslands in the Alps; however, no significant effect of cattle density on carabid species richness, assemblage structure or functional traits was detected [47].

It should be noted that a more pronounced effect of the wisent’s influence was demonstrated in the enclosures. In the free-ranging population, despite the fact that meadows (the main food source for European bison) were analyzed, no such effect was found. The reason for this was probably a small herd that was released into the wild, the impact of which was imperceptible. Moreover, the intense grazing on meadows by the free-ranging population does not correspond to the intense grazing in the enclosures, where animal density is not natural. Nevertheless, an effect can also be expected in larger free-ranging populations. European bison do not use the entire area of forest complexes evenly [48,49], and herds of cows tend to routinely use some particular parts of the available space. It can, therefore, be speculated that such an effect may be more pronounced where animals congregate. The selection of habitats depends on the location of the population and can vary significantly [50]. Attention should also be paid to the fact that the use of forest habitats does not necessarily have a nutritional dimension, as European bison may use sites for other purposes, e.g., for rumination [51]. The structure of stands may also play a role in the intensity of grazing, as has been demonstrated for other grazers [52,53]. Therefore, capturing the effect of grazing on carabid fauna may be difficult to achieve.

Because only three European bison populations have been studied for a relatively short time, the validity of the results may be limited. In the Augustowska forest the population was studied in the first year after reintroduction of the bison (second year of grazing) and a longer time of grazing may result in more clear effects. Repeated studies on the study sites as well as additional study sites might help to corroborate our findings. Besides, pitfall traps, as used in our study, contain a bias because results depend on both abundance and activity of the beetles. As a consequence, the results may be skewed towards larger species [54,55].

As shown by studying carabid beetle assemblages in the olive agroecosystem [56], agricultural practices’ interactions with the background of the respective ecosystem is a complex issue. This is also true when using grazing by European bison as a nature conservation measure. In this context, it should also to be considered that grazing may also have a negative impact. For example, studies have indicated that grazing has a negative impact on small mammals [57]. The effects of grazing on organic content of the soil are also very complex and grazers might be managed differently to help mitigate greenhouse gas emissions [58]. Therefore, different variables such as timing, duration, cessation and intensity of grazing have to be taken into account, because they greatly modify the structure of the respective grassland [13]. According to researchers [59] who studied grazing by Polish Konik horses, grazing intensity should be adjusted annually to balance vegetation development.

## 5. Conclusions

Our study indicates an impact of increased European bison grazing activity on carabid beetles. Since the study revealed only a weak relation between the intensity of European bison grazing and carabid assemblage structure, but a stronger relation with functional traits of the assemblages, it indicates that using European bison grazing as a method of ecological engineering in the context of nature conservation may have more potential to regulate ecosystem properties and functions than to conserve specific species or species assemblages of carabid beetles. However, in order to apply grazing in a reasonable manner, different variables such as timing, duration, cessation and intensity of grazing have to be taken into account.

## Figures and Tables

**Table 1:** Grazing intensity in 2018–2019 and numbers of collected individuals and species of carabid beetles in 2017 and 2019 at the study sites in Augustowska Forest.

Study Site	Grazing Intensity (2018–2019)	Individuals 2017	Species 2017	Individuals 2019	Species 2019
A212	high	874	43	2048	57
A78	low	1393	41	745	42
A132	low	1094	46	942	41
A169	none	970	31	1598	32
A227	none	1025	36	723	35

**Table 2:** Redundancy Analysis (RDA) of the carabid beetle assemblages at the study sites in Augustowska Forest: results of Monte Carlo permutation tests of the environmental variables using automatic forward selection of variables (reduced model). Variable “2018” was not added to the model due to collinearity.

Variable	Lambda-A	F	*p*
2017	0.09	0.77	0.614
Intensity	0.08	0.70	0.642

**Table 3:** Redundancy Analysis (RDA) of the functional traits of the carabid beetle assemblages at the study sites in Augustowska Forest: results of Monte Carlo permutation tests of the environmental variables using automatic forward selection of variables (reduced model). Variable “2018” was not added to the model due to collinearity.

Variable	Lambda-A	F	*p*
Intensity	0.14	1.26	0.288
2017	0.07	0.61	0.613

**Table 4:** Grazing intensity and numbers of collected individuals and species of carabid beetles in 2018 at the study sites in the Kobiór Forest district and the Niepołomice Forest District.

Area	Area Type	Study Site	Grazing Intensity (2018)	Individuals 2018	Species 2018
Jankowice	meadow	MG	low	340	20
		MN	none	308	27
	Forest	JG1	low	1836	19
		JG2	low	986	11
		JN1	none	958	8
		JN2	none	689	15
Niepołomice	Forest	NG1	high	1715	24
		NG2	high	507	23
		NN1	none	1558	21
		NN2	none	718	19

**Table 5:** Canonical Correspondence Analysis (CCA) of the carabid beetle assemblages of the study sites in the Kobiór Forest district and the Niepołomice Forest District: Results of Monte Carlo permutation tests of the environmental variables using automatic forward selection of variables (reduced model). Variables “Meadow” and “Niepołomice” were not added to the model due to collinearity.

Variable	Lambda-A	F	*p*
Forest	0.81	6.58	0.029
Jankowice	0.23	2.14	0.013
Intensity	0.19	1.95	0.076

**Table 6:** Redundancy Analysis (RDA) of the functional traits of the carabid beetle assemblages of the study sites in the Kobiór Forest district and the Niepołomice Forest District: Results of Monte Carlo permutation tests of the environmental variables using automatic forward selection of variables (reduced model). Variables “Meadow” and “Niepołomice” were not added to the model due to collinearity.

Variable	Lambda-A	F	*p*
Forest	0.76	25.45	0.029
Intensity	0.10	4.59	0.034
Jankowice	0.04	2.68	0.130

## Data Availability

The data presented in this study are available on request from the corresponding author.
